# Eruptive widespread crateriform papulonodules and back pain: An interesting case of Langerhans cell histiocytosis?

**DOI:** 10.1016/j.jdcr.2025.03.032

**Published:** 2025-04-17

**Authors:** Cruz Riley, Behnam M. Abdollahi, Aparche B. Yang

**Affiliations:** a Kaiser Permanente Bernard J. Tyson School of Medicine, Pasadena, California; b Department of Pathology, Southern California Permanente Medical Group, Irvine, California; c Department of Dermatology, Southern California Permanente Medical Group, Irvine, California

**Keywords:** BRAFV600E, Langerhans cell histiocytosis

## Introduction

Langerhans cell histiocytosis (LCH) is a neoplastic histiocytic disorder characterized by aberrant proliferation of myeloid dendritic cells due to a mutation in the mitogen-activated protein kinase/extracellular signal-regulated kinase pathway. LCH is exceptionally rare in adults and can arise in any tissue creating a diagnostic challenge. Here we describe the case and differential diagnosis of adult-onset multisystem LCH presenting as new skin lesions and back pain.

## Case

**Fig 1:**
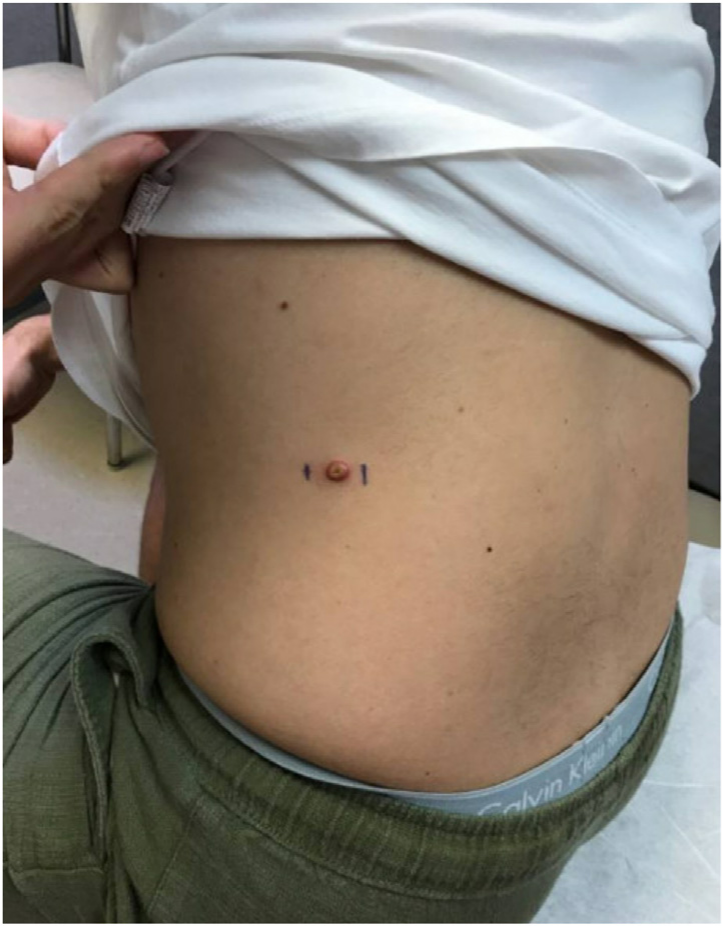
Image of ∼5 mm *pink* crateriform papulonodule with central dried tough pustule located on the left flank.

**Fig 2:**
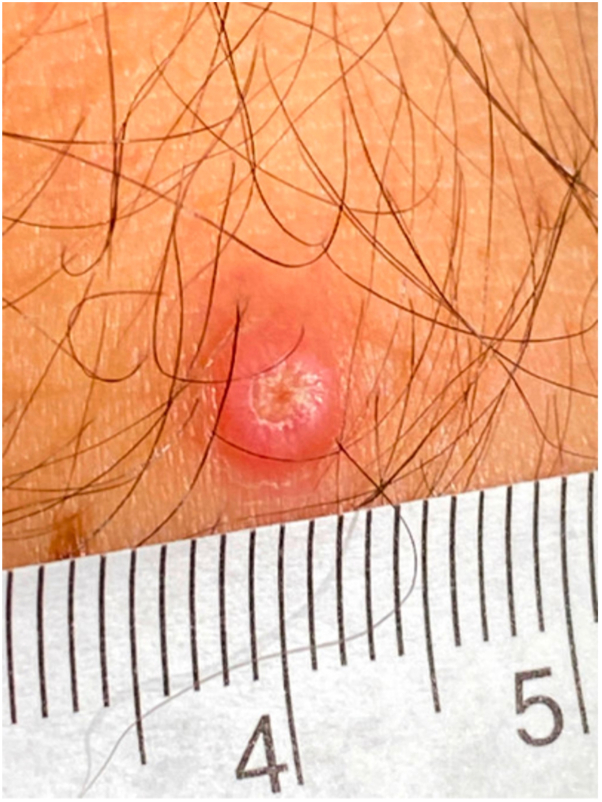
Image of ∼5 mm *pink* crateriform papulonodule with central dried tough pustule located on the right forearm.

A 30-year-old male presented to dermatology for evaluation of several tender and pruritic lesions across the scalp, trunk, and extremities. The lesions developed suddenly 2 months after a camping trip in rural Nevada. Review of systems revealed new, concomitant low back pain worsened by movement. Patient denied musculoskeletal weakness, prodromal symptoms, or new sexual partners. Past medical history, baseline metabolic labs, and infectious investigation were unremarkable. Skin exam showed multiple ∼5 mm pink crateriform papulonodules, many with a central dried tough pustule, on the scalp, trunk, left upper and lower extremities, and right forearm (Figs 1 and 2). There was no involvement of the nails, mucosa, or groin.

**Fig 3:**
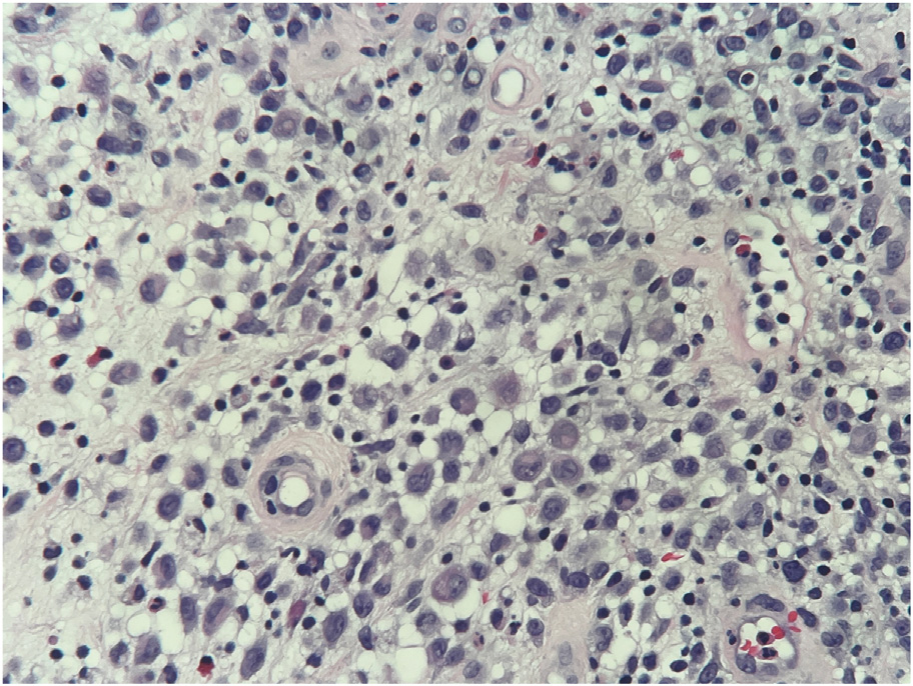
Photomicrograph of a hematoxylin-eosin–stained section reveals dermal infiltrate primarily composed of enlarged cells with reniform and abundant cytoplasm. Admixed eosinophils and neutrophils are also identified (hematoxylin-eosin, original magnification ×200).

**Fig 4:**
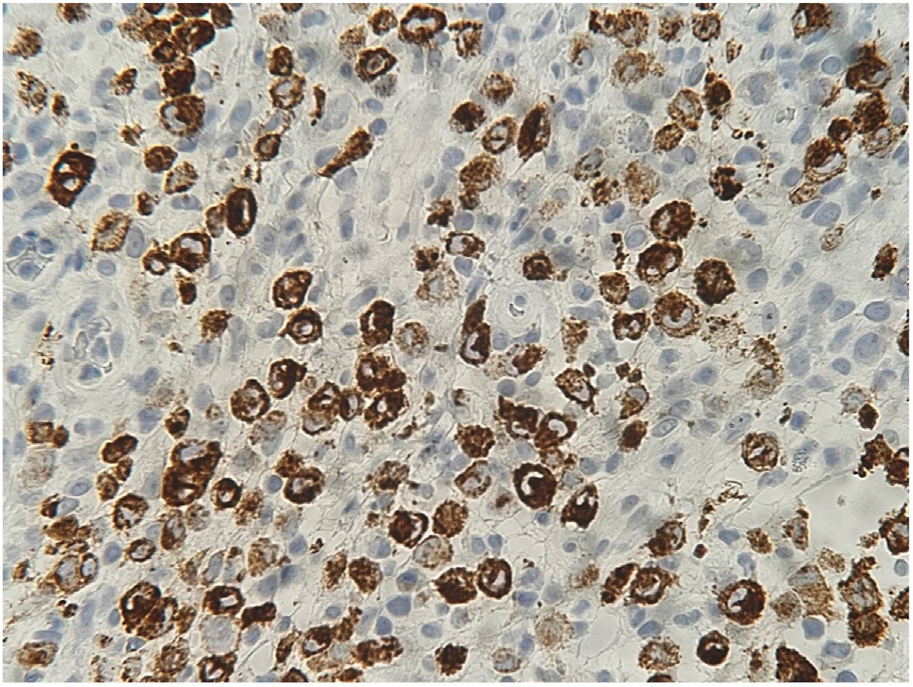
Photomicrograph of dermal infiltrate with positive staining for CD207 (Langerin) (hematoxylin-eosin, original magnification ×400).

Skin biopsy showed histologic and immunohistologic features consistent with LCH, characterized by a proliferation of histiocytes with reniform nuclei (Fig 3) and positive staining for S100, CD68, CD1a, and CD207 (Langerin) (Fig 4). Infectious workup was negative. BRAF mutation analysis, bone marrow biopsy, and peripheral blood smear were also negative. A staging positron emission tomography (PET) scan revealed uptake at the mediastinum, liver, and thoracic spine. A computed tomography-guided biopsy of the T10 vertebral body confirmed clusters of Langerhans cells, thus favoring a primary LCH process as opposed to a reactive process. The cutaneous lesions cleared with topical clobetasol 0.05% ointment. Systemic treatment with cytarabine and cladribine was considered; however, repeat PET scan approximately 4 months after initial diagnosis showed spontaneously remitted LCH. Thus, hydroxyurea was initiated to deepen remission.

## Discussion

LCH is a histiocytic disorder characterized by aberrant proliferation of myeloid dendritic cells due to an activating mutation in the mitogen-activated protein kinase/extracellular signal-regulated kinase pathway.^1^ BRAFV600E, the most common of these mutations, is associated with younger age at diagnosis and higher prevalence for multisystem disease and skin involvement.^2^ BRAFV600E mutation in adults with LCH is correlated with a higher incidence of secondary primary malignancies (SPMs), most commonly a hematologic malignancy.^3^ A recent population-based study found SPMs to be the most common cause of death in adult LCH, while in pediatric LCH, the most common cause of death was infections.^4^ Estimated annual incidence of LCH is 4.6 cases per 1 million children under 15 years of age.^1^^,^^5^ Adult-onset LCH is more rare; however, incidence estimates are less precise likely due to its variable clinical presentation.^5^ Interestingly, skin or bone-limited adult-onset LCH has been observed to spontaneously remit in a handful of case reports, further complicating incidence estimates. Spontaneous remission of multisystem adult-onset LCH is even more scarcely observed.

Clinically, LCH can present in a variety of single and multi-organ constellations creating a diagnostic challenge. In single-system LCH, the lungs are the most common site of involvement. In multisystem LCH, bone and skin are the most involved although any organ system can be affected.^2^ Cutaneous LCH has a variety of nonspecific morphologies, including erythematous papulonodules, nonhealing ulcers, and rashes resembling dermatitis, urticaria, and less commonly vitiligo.^5^ Thus, diagnosis of cutaneous LCH relies upon skin biopsy. Microscopically, LCH is characterized by the proliferation of histiocytes with reniform nuclei and positive immunohistochemical staining for S100, CD1a, and CD207 (Langerin).^2^ CD68 staining is variable.^1^

Molluscum contagiosum, cutaneous cryptococcus, and variants of multiple keratoacanthoma should be included in the differential due to their morphologic similarity with the patient’s lesions. Molluscum contagiosum, a common poxvirus, classically presents as umbilicated papules. Molluscum contagiosum has a propensity to develop in the first decade of life; however, cases do occur in adults and are usually sexually transmitted, particularly in the setting of immunocompromise.^6^ Cutaneous cryptococcus is a skin infection caused by encapsulated fungi. Similar to molluscum contagiosum, it typically presents as dome-shaped papules with central umbilication. It is 1 of the most common opportunistic fungal skin infections to affect HIV-positive patients worldwide but also may infect immunocompetent hosts.^7^ Our patient is HIV-negative. Keratoacanthomas are rapidly growing and relatively benign tumors which present as dome-shaped nodules each with a central keratin crater.^8^ Lesional biopsy is critical for diagnostic certainty and appropriate management.

The patient’s travel history to Nevada raised the possibility of coccidioidomycosis. Also known as “Valley Fever,” coccidioidomycosis is a primary lung disease that results from infection by fungus endemic to the southwestern United States, including Nevada. Cutaneous presentations are typically evidence of disseminated disease, but primary cutaneous coccidiomycosis due to direct inoculation skin injury has been rarely observed.^9^ Cutaneous coccidioidomycosis has a wide variety of presentations. Diagnosis is based on Periodic acid-Schiff-positive spherules on tissue histopathology.

Mpox, formerly known as monkeypox, is a poxvirus endemic to rural areas of West and Central Africa. In May 2022, an mpox outbreak occurred in the United States around the same time as our patient’s initial presentation. Morphologically, mpox appears as umbilicated papules with central ulceration.^10^ This diagnosis was ruled out in our patient with a negative MPox/Orthopox polymerase chain reaction test.

## Conclusion

This case demonstrates a challenging presentation of eruptive, spontaneously remitting adult-onset LCH. Despite its rarity in adults, LCH should be considered in cases of eruptive widespread papulonodules with unexplained musculoskeletal symptoms. Diagnosis of LCH, especially in BRAFV600E mutation-positive patients, warrants thorough staging with comprehensive imaging, bone marrow biopsy, and peripheral blood smear to exclude secondary organ involvement and, if relevant, SPMs.

## Conflicts of interest

Drs Yang and Abdollahi are salaried partners of the Southern California Permanente Medical Group. Dr Riley has no conflicts of interest to declare.
